# Supplementary material for: Immune checkpoint inhibition perturbs neuro-immune homeostasis and impairs cognitive function
Source: J Exp Clin Cancer Res. 2025 Jul 2;44:183. doi: 10.1186/s13046-025-03442-3 (PMC12220143; doi:10.1186/s13046-025-03442-3)
Supplement: Supplementary file 1 — Supplementary Material 1 [file 13046_2025_3442_MOESM1_ESM.docx]

**SUPPLEMENTAL INFORMATION**

*Immune Checkpoint Inhibition Perturbs Neuro-immune Homeostasis and Impairs Cognitive Function*, by Ifejeokwu and Do, *et al.*

**
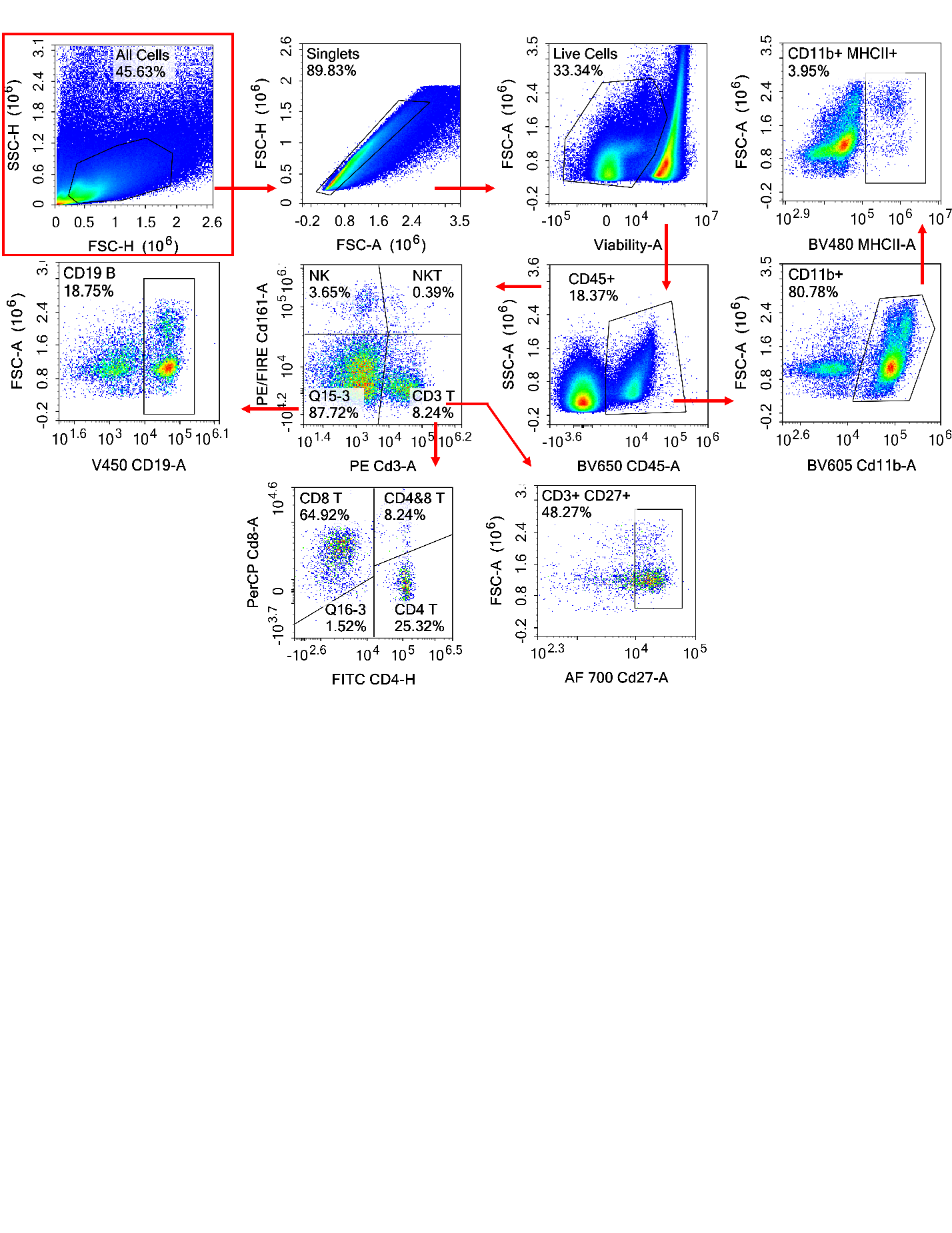
Suppl. Fig. S1 (SF1)**

**Supplemental Figure S1. Gating strategy for immunophenotyping cells in the brain.** Sequential gating strategy to various immune cell subsets. First, doublets were excluded using FSC-A vs FSC-H plot. Then, CD45 was used to gate all immune cells among the live population. CD45+ gate was further used to gate NK, T, and NK T cells by plotting CD161 and CD3. Memory T cells were identified by gating on cells for CD27 expression. T cell subtypes were identified by plotting CD8 by CD4. Non-NK and T cell gates were used to identify CD19+ B cells. CD11b marker was used to gate myeloid populations. Then, the CD11b+ gate was used to further distinguish myeloid populations expressing MHCII.

**
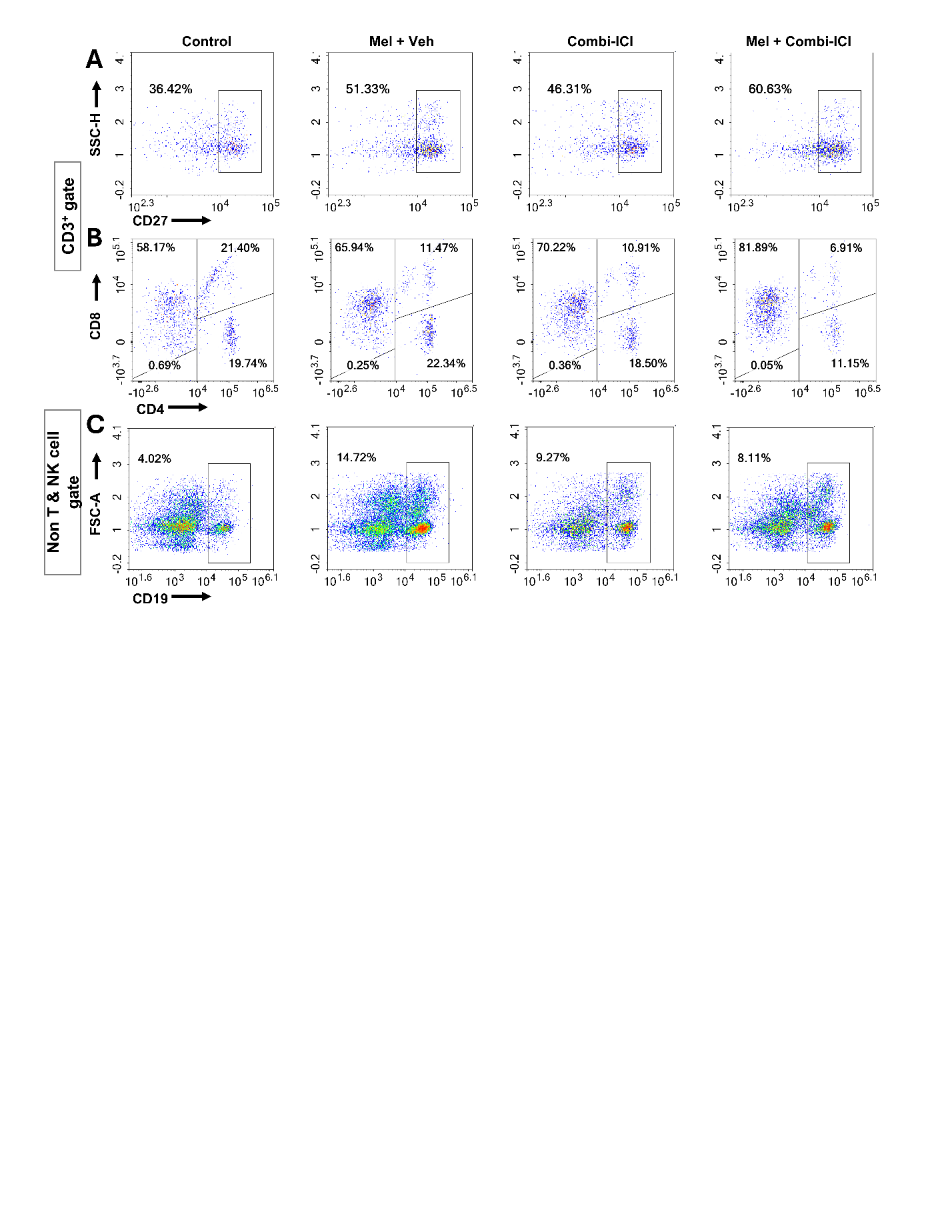
Suppl. Fig. S2 (SF2)**

**Supplemental Figure S2. Gating strategy for immunophenotyping T and B cells.**
**(A)** Representative dot plots of CD27+ CD3+ T cell gating.
**(B)** Representative dot plots of CD8+ CD4+ T cell gating.
**(C)** Representative dot plots of CD19+ B cell gating.

**Suppl. Fig. S3 (SF3)**

**
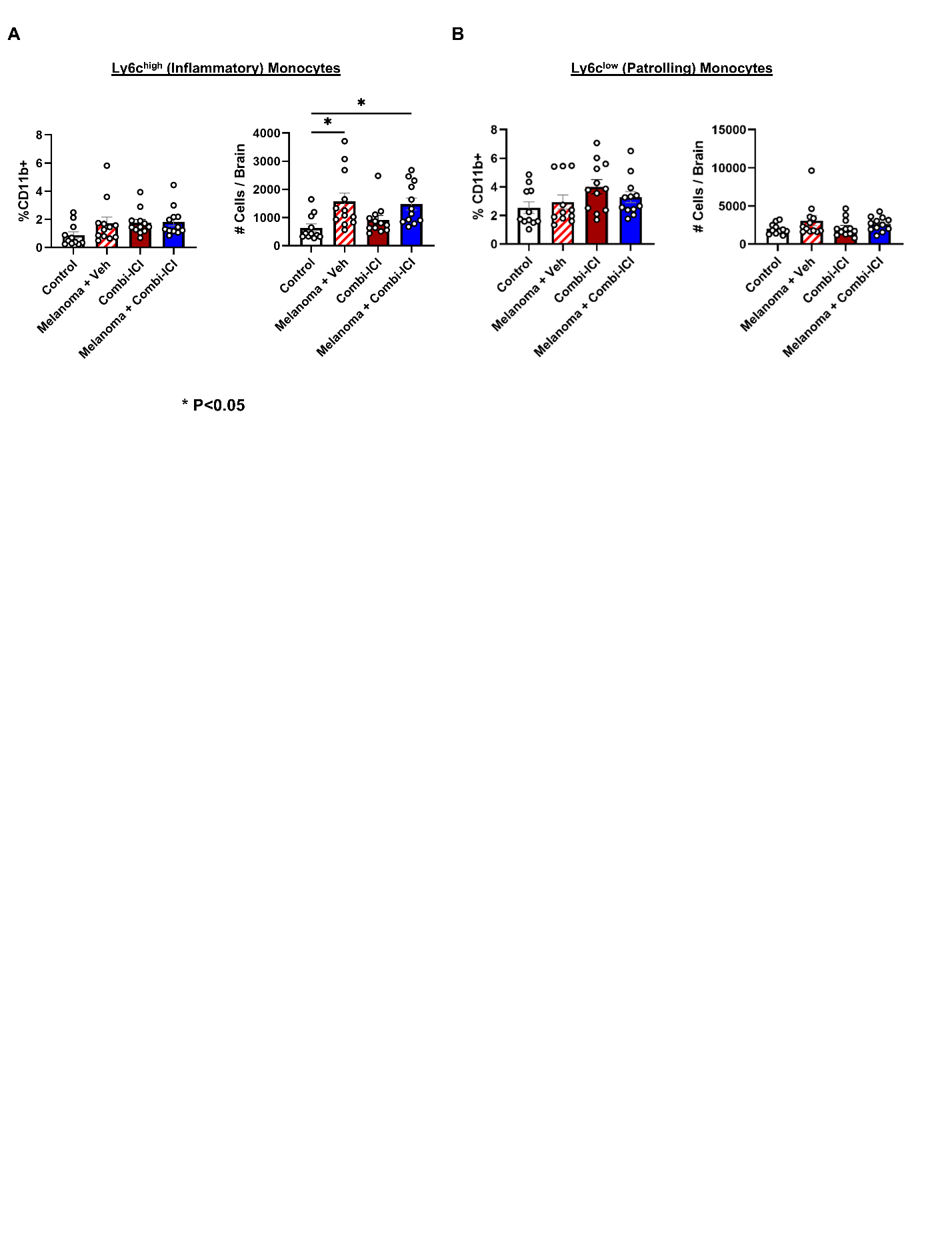
**

**Supplemental Figure S3. CD45+ CD11b+ monocytes in the brain.**

**(A & B)** Frequencies (% of CD11b+) and number of Ly6c^high^ (**A**) and Ly6c^low^ cells (**B**).

**Suppl. Fig. S4 (SF4)**

**
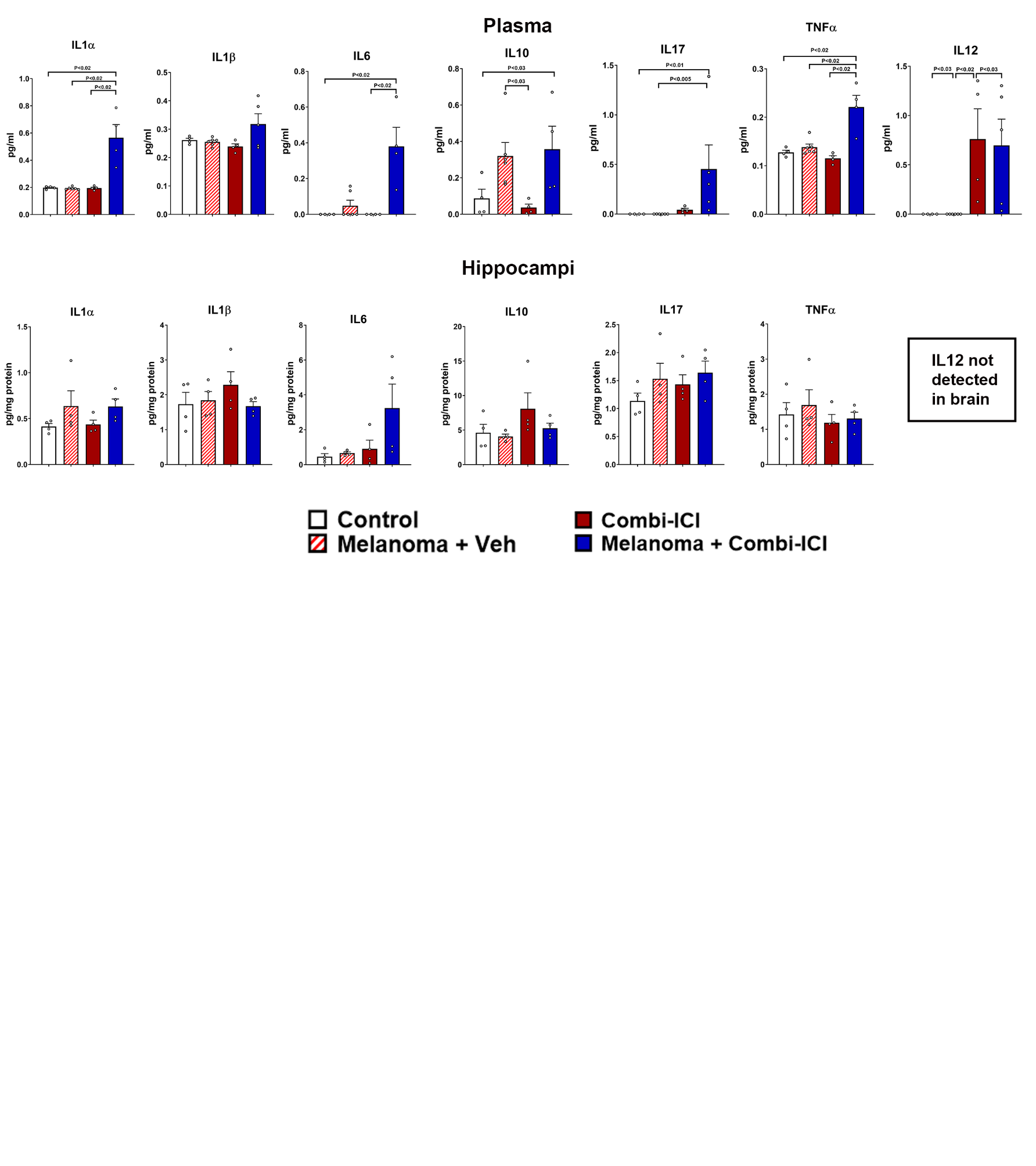
**

**Supplemental Figure S4. Plasma and brain cytokines.**Cytokine levels plasma and hippocampi. For Hippocampi, detected quantity was normalized to the total protein content of dissected tissue. Data are presented as mean ± SEM (N=4-6 mice/group). *P* values were derived from Kruskal-Wallis test.

**Suppl. Fig. S5 (SF5)**


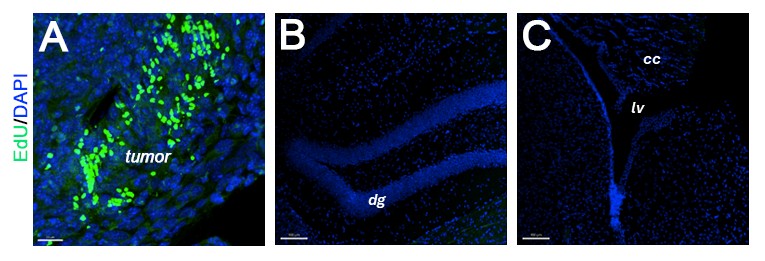


**Supplemental Figure S5. Absence of EdU+ D4M-UV2 cells in the brain.** D4M-UV2 cells were treated with 50 µM EdU for 30 minutes before injecting for cell tracking. At 10 days post-cancer induction, mice were euthanized. Paraformaldehyde-fixed brains and tumors were cryosectioned to collect 30 μm thick tumor and coronal brain sections for a Click chemistry-based EdU staining. **(A)** Representative image of EdU staining (Green, DAPI nuclear counterstain, Blue) within the perimeter of a resected tumor. Scale bar = 20 µm. **(B-C)** Corresponding representative images from brain regions of same host mouse from **(A)** showing absence of EdU+ green cells in the hippocampus **(B)** and lateral ventricle **(C**). Scale bars 20 µm **(A)**, and 100 µm (**B-C**). N=3 mice.

**SUPPLEMENTAL MATERIALS AND METHODS**

**Correspondence**

Further requests for information about resources and reagents should be directed to and will be fulfilled by the corresponding authors**.**

**Materials Availability**

This study did not generate any new unique reagents.

**Data and Code Availability**

- All data reported in this paper will be shared upon request.
- This paper does not report the original code.
- Any additional information required to reanalyze the data reported in this study is upon request.

**EXPERIMENTAL MODEL AND ANIMAL DETAILS**

**Mice**

All animals used in this study were in accordance with the National Institute of Health (NIH) guidelines and approved by the Institutional Animal Care and Use Committee (IACUC) at University of California, Irvine. Wild-type male mice (C57BL/6J, Jackson Laboratory RRID:IMSR_JAX:000664), aged 10-12 weeks were group housed (2-4 mice per cage) in standard conditions (20 °C±1 °C; 70% ± 10% humidity;12h:12h light and dark cycle) and given a standard rodent chow diet (Envigo Teklad 2020X) by the University Laboratory Animal Resources (ULAR).

**D4M-3A.UV2 Melanoma induction and tumor measurement**

D4M-3A.UV2 mouse melanoma cells were cultured in monolayer with Gibco^TM^ DMEM (1X) + GlutaMAX^TM^-I (FisherSci, Cat. 10-569-010) supplemented with 10% Gibco^TM^ FBS (FisherSci, Cat. 10-082-147) under 37°C/5% CO_2_ conditions. On the day of tumor induction, D4M-3A.UV2 cells were trypsinized in Gibco™ TrypLE™ Express Enzyme (FisherSci, Cat. 12-605-010) and centrifuged (300g, 5 minutes). Trypsin was neutralized with DMEM + 10% FBS, washed, and centrifuged (300g, 5 minutes) to remove excess TrypLE. Cells were resuspended in Gibco™ Hibernate™-A Medium (FisherSci, Cat. A1247501) and kept on ice until tumor induction. 1X PBS (FisherSci, Cat. 14-190-144) was used to spray down the injection area (left flank) to help the researcher detect the injection site without interference from the fur. Each mouse subject received an intradermal injection dose of 1 x 10^6^ D4M-3A.UV2 cells in 0.2mL Hibernate Medium (FisherSci) on the left flank using a 31 Gauge insulin syringe (BD Sciences, Cat. 328411-1) until a small bleb appeared underneath the skin. The bleb indicates that the cells were successfully injected under the dermis. Mice were monitored every other day for tumor growth. Tumor growth was measured using an electronic caliper along the tumor's horizontal and vertical axis. The maximum diameter was determined as the larger diameter between vertical and horizontal measurements. The tumor volume was calculated using the formula 0.5 x *a x b*^2,^ where *a* is the minimum diameter and *b* is the maximum diameter. Mice were treated with Immunotherapy when the tumor volumes were approximately 20-30 mm^3^ (7 days post-tumor induction).

**EdU pulsed D4M-3A.UV2 Melanoma for Cell Tracking**

D4M-3A.UV2 mouse melanoma cells were cultured and injected as described above. Prior to trypsinizing cells on tumor induction day, D4M-3A.UV2 were treated with Click-iT™ EdU (Invitrogen, Cat. C10350) using a final concentration of 50 µM for 30 minutes. Animals received no immunotherapy treatment and tumors engrafted for 10-12 days before tissue collections. Brains and tumors were collected for cell tracking.

**Combined Immunotherapy of anti-CTLA-4 and anti-PD1**

After one week of melanoma induction, mice in the treatment groups received the combined Immunotherapy of anti-CTLA-4 (BioXcell, Cat. BP0032 RRID: AB_1107598) and anti-PD 1 (BioXCells, Cat. BP0273 RRID: AB_2687796). Anti-CTLA-4 and anti-PD1 were diluted with InVivoPure pH 6.5 Dilution Buffer (BioXCells, Cat. IP0065) to 1 mg/mouse and 200µg/mouse, respectively. Anti-CTLA-4 was given intraperitoneal injections 3 times/week on Monday, Wednesday, and Friday, and anti-PD1 2 times/week on Monday and Thursday continuously for 3 weeks. Control mice that did not receive the combined ICI treatments were injected with isotype-matched control (Isotype control for CTLA-4: Armenian Hamster IgG, BioXcell, Cat. BP0091, RRID: AB_1107773; Isotype control for PD1: Rat IgG2a, BioXCell, Cat. BP0089, RRID: AB_1107769) following the same injection schedule as the mice in the treatment groups.

**Open Field Test and Object Location Memory**

Four weeks after the initiation of the combined ICI treatment, behavior tests were administered to determine the impact of the combined ICI treatment on cognitive function. Open field test (OFT) and object location memory (OLM) test were performed. For both tasks, the experimental setup includes a strictly controlled room with appropriate lighting (50-70 lux), four square open-field arena boxes (30 x 30 x 30 cm), camera recording equipment (Noldus), and tracking software (EthoVision XT 17, Noldus). The OFT task evaluated an animal's preference to stay in the open field (30% of the central zone) or the shaded outer edge (70% of the outer edge of the arena) and was performed on the first day of habituation of the OLM task. The time the mice spent either in the open field Or the shaded area of the arenas was quantified to assess their exploratory behaviors. Spatial recognition and memory were evaluated using the OLM task, which highly depends on an intact and functioning hippocampus (2,3). The OLM tasks were conducted as reported previously (1, 7, 10). Briefly, the mice were habituated with the testing room and open field arena boxes (30 x 30 x 30 cm) that had blue tape down the inside wall of the arena box. The boxes had a thin layer of bedding, and no toys were present; the mice were allowed to familiarize themselves with the testing environment for 10 minutes per run on 3 consecutive days (with OFT data collected on the first habituation day). During test day, the familiarization phase was conducted by allowing the mice to explore two identical plastic toys that were magnetically secured in place 16 cm apart from each other for 5 minutes (familiar phase). After the 5-minute of familiarization, the mice were returned to the home cage for 5 minutes while the objects were cleansed with 10% ethanol and thoroughly dried. One object was then moved to a new location inside the box (novel place), 16 cm from the opposite corner of the other toy, which remained in its former spatial location (familiar place). Mice were returned to arena boxes and allowed to explore for 5 minutes. Following the test phase, all mice were returned to their home cage. Mice behavior from all phases was recorded using the Noldus camera system. The "head direction to zone" function from Ethovision software was utilized to track the mouse behavior and record their exploration time. Furthermore, to ensure unbiased analysis, time spent interacting (nose within 2 cm) with familiar versus novel place objects was scored by researchers blind to the experimental conditions. The discrimination index (DI) was calculated for each animal using the equation: ([Novel object or location exploration time/Total exploration time] – [Familiar object or location exploration time/Total exploration time]) × 100.

**Light-Dark Box (LDB)**

After the OLM test day, anxiety-like behavior in mice was evaluated by performing the light-dark box (LDB) test using established methods as reported previously (4, 7, 8). The LDB arena consisted of a dark compartment (15 x 10 x 27 cm, 4 lux) connected to a light compartment (30 x 20 x 27 cm, 915 lux) via a small opening (7.5 x 7.5 cm). This task juxtaposes a mouse's inclination to explore new environments with their level of anxiety to be in a well-lit space. Mice were allowed to explore the LDB arena for 10 minutes; the time spent in each compartment and the number of transitions between the light and dark compartments were recorded.

**Fear Extinction Memory**

After OFT and OLM cognitive task, we performed fear extinction behavior reliant on hippocampal function to determine if the combined treatment of anti-CTLLA-4 and anti-PD1 affects amygdala-hippocampal circuit-dependent fear conditioning and memory consolidation process (5, 9). The experimental setup includes a behavioral conditioning chamber (17.5 × 17.5 × 18 cm, Coulbourn Instruments) with steel shock floors (3.2 mm diameter slats, 8 mm spacing), a waste collection tray sprayed with 10% vinegar, dim light, and a well established shock-tone system connected to the chamber (5, 9). During the first habituation day (Day 1), mice were allowed to habituate to the chamber for two minutes. Then, three pairs (evenly spaced at two-minute intervals) of auditory conditioned stimulus (CS; 16 kHz tone, 80 dB, lasting 120 sec) co-terminating with a mild foot shock unconditioned stimulus (US; 0.6 mA, 1 sec) were given. Twenty-four hours later, on the subsequent 3 days of extinction training phase (Days 2-4), mice were initially habituated to the same contextual environment (dim light and vinegar odor) for two minutes before being presented with 20 non-US reinforced CS tones (16 kHz, 80 dB, lasting 120 sec, at 5 sec intervals). Twenty-four hours later (Day 5), fear testing was administered by presenting the mice with only three non-US reinforced CS tones (16 kHz, 80 dB, lasting 120 sec) at two-minute intervals in the same context (hippocampal-dependent). At 72 hours after completion of hippocampal-depedent extinction test, an mPFC-dependent extinction test was administered using three non-US reinforced CS tones (16 kHz, 80 dB, lasting 120 sec) at two-minute intervals in a different environment, including a white acrylic plate as a floor, new odor cue (10% almond in water), and an additional house light. Animals' freezing behavior was recorded using a ceiling-mounted camera in the FE test chamber and by an automated, video-based motion measurement program (FreezeFrame, Coulbourn Instruments). FreezeFrame algorithms calculated a motion index for each video frame, with higher values representing greater motion. To prevent biased analysis, an investigator blinded to the experimental groups set the motion index threshold representing immobility for each animal based on identifying a trough separating low values during immobility and higher values associated with motion. Freezing behavior was defined as continuous bouts of one second or more of immobility. The percentage of time each mouse spent freezing was then calculated for the final day of extinction test.

**BrdU Preparation and Administration**

One week after the last dose of the last immunotherapy injections, mice were administered with BrdU (5-Bromo-2′deoxyuridine, 50 mg/kg, I.P., once daily for 6 days, Sigma, Cat. B5002) to evaluate the impact of ICI treatment on *in vivo* neurogenesis. BrdU was pre-weighed into 75mg in six carefully labeled 50mL Falcon tubes (FisherSci, Cat. 14-432-22) and kept at -20^o^C until use. On the day of injection, one falcon tube was thawed to room temperature (RT) before processing. BrdU was dissolved in 30mL of 1X PBS (100mM, pH=7.6, FisherSci) and kept in a warm water bath (55-60°C) for 20 minutes. The solution was thoroughly vortexed until all visible particles were dissolved. Additionally, BrdU solution was filtered through Whatman® Puradisc 25 syringe filters (Sigma, Cat. WHA67502502) to remove any remaining undissolved BrdU particles. The solution was allowed to cool down to RT before injecting the mice with the dosage described previously (8).

**Brain Tissues and D4M-3A.UV2** **Tumor Collection for Immunohistochemistry**

Mice were deeply anesthetized by inhaling 1.8% isoflurane v/v (Dechra) and euthanized via intracardiac perfusion by flushing ice-cold 1X PBS + 10U/ml heparin (Sigma, pH=4) in the left atrium until the venous outflow was clear. Perfusion with 1X PBS + 10U/ml heparin was quickly replaced with 4% PFA (Sigma) until the mouse body turned stiff, and whole brains and tumors were immediately extracted and soaked in vials filled with 4% PFA (Sigma) overnight. Whole brains and tumors were switched to store in 1X PBS-0.05% Sodium Azide (Sigma-Aldrich Cat. S2002, pH 7.4. Whole brains were dehydrated by submerging in a sucrose concentration gradient (10% to 30% w/v, Sigma, Cat. S7903, pH=7.4) to avoid the formation of crystals during cryo-sectioning. After cryoprotection, whole brains and tumors were embedded in O.C.T compounds (VWR, Cat. 25608903) to support and stabilize the brains during sectioning. Each brain and tumor were sectioned at 30 µm thickness (coronal) and stored as floating tissues in 24-well plates in 1X PBS-0.05% Sodium Azide (Sigma-Aldrich, pH 7.4) at 4^o^C until next steps.

**Immunohistochemistry**

Floating brain sections (N=4-8 brains/group, 2-3 sections/ brain) with visible hippocampi and tumors (N=3 tumors, 3 sections/tumor) were chosen for immunohistochemistry (IHC). Primary antibodies include Rat anti Mouse CD68 (1:500, Bio-Rad, Cat# MCA1957; RRID: AB_322219 ), Rabbit anti IBA1 (1:500, Wako, Cat# 019-19741; RRID: AB_839504), Mouse anti NeuN (1:500, Millipore, Cat# MAB377, RRID:AB_2298772), Rabbit anti cFos (1:500, Abcam, Cat# ab190289, RRID:AB_2737414), Mouse anti GFAP (1:500, FisherSci, Cat# MA5-12023, RRID:AB_10984338), Rat anti MBP (1:500, Millipore, Cat# MAB386, RRID:AB_94975), Mouse anti Synaptophysin (1:1000, Sigma, S5768), Rat anti BrdU (1:150, Abcam, Cat# ab6326, RRID:AB_305426), Rabbit anti NeuN (1:500, Millipore, Cat# MABN140, RRID:AB_2571567) and Mouse anti PSD-95 (1:1000, FisherSci, Cat# MA1-045; RRID: AB_325399). Secondary antibodies include Goat anti Rat AF 647 (1:1000, Abcam, Cat# ab150159; RRID: AB_2566823), Goat anti Rabbit AF 488 (1:500, FisherSci, Cat# a11008; RRID: AB_143165), Donkey anti Mouse AF 488 (1:350, FisherSci, Cat# A-21202, RRID:AB_141607), Donkey anti Rabbit AF 568 (1:350, Abcam, Cat# ab175470, RRID:AB_2783823), Goat anti Mouse AF 568 (1:500, Abcam, Cat# ab175473, RRID:AB_2895153), Goat anti Rat AF 568 (1:1000, Abcam, Cat# ab175476, RRID:AB_2813739), Click-iT™ AF488 azide (1:500, Cat# C10350), and Goat anti Mouse AF 647 (1:1000, Abcam, Cat# ab150115; RRID: AB_2687948).

For staining, tissue sections were first washed 3 times in phosphate-buffered saline (1X PBS, 100mM, pH 7.4, FisherSci) and, when necessary, antigen retrieval was performed using citrate buffer incubation (10mM, pH 6.0 with 0.05% Tween-20, Sigma) 70°C for 45 minutes. Tissues were then incubated in borate buffer (pH 8.5, 100mM, Sigma, Cat. B0394) for 10 minutes, followed by three 5-minute washes in 1X PBS. Tissues were then blocked in serum (10% normal goat or donkey, NGS or NDS) in PBS with 0.01% Triton X-100 (NDS, Jackson ImmunoResearch Labs Cat# 017-000-121, RRID: AB_2337258; NGS, Jackson ImmunoResearch Labs Cat# 005-000-121, RRID:AB_2336990). Following an hour of incubation in the blocking solution, the tissues were incubated in primary antibodies for glial or neuronal components. Primary antibodies were prepared in 0.01% Triton X-100 (Sigma) 3% NGS or NDS in PBS and incubated for at least 12 hours at 4°C in a shaker incubator (Enviro-Genie Scientific). The tissues were washed in 1X PBS (3 times) and then incubated in the secondary antibodies for one hour. Finally, sections were counterstained with DAPI nuclear dye in PBS for 15 min (1 µmol/L, FisherSci, Cat# D1306, RRID:AB_2629482). Finally, sections were washed in PBS and then mounted on superfrost slides (FisherSci) using Antifade mounting medium (VectaShield Cat# H-1000-10).

**Microscopy and 3D Algorithm-based Volumetric Quantification**

Laser-scanning confocal microscope (Nikon Eclipse Ti2 AX) was used to image the immunostained brain sections at high resolution (2048p, 24 µm thick z-stacks with 0.5 µm per z-stack). Acquisition of confocal z stacks, deconvolution, and 3D algorithm-based volumetric quantification of immunofluorescent punta was carried out as described in detail previously (10). 3D volume surfaces were created and quantified for each antigen of interest. Dual IHC stains such as CD68/IBA1, and co-localization volumetric between the 3D surfaces of the two markers were determined and automatedly quantified by Imaris (Oxford instruments). All images for each IHC stain were uniformly applied with the same parameters to obtain unbiased analyses.

**Cell Isolation from Brain Tissue for Flow Cytometry**

72 hours following the last ICI injection, mice brains were collected. Mice were placed in a CO2 chamber until respiration stopped and immediately perfused (intracardial) with 25ml of 1X PBS (Gibco, Cat# 10010-023) containing 2mM EDTA (Invitrogen, Cat# 15575-038) using 50ml syringes (BD Biosciences, Cat# 309653) and 21 ¾ gauge butterfly needle (Abbott Lab, Cat#4492). Brains were dissected and placed in 1 ml of AIMV media (Thermo Fisher, Cat. 12055083) in 15 ml conical tubes on ice (Falcon, Cat# 352097). The brain tissue was homogenized until all large chunks were broken apart using a 15 ml homogenizer (VWR, Cat# 47732-446). Samples were then transferred to a 15 ml vented cap conical tube (Celltreat, Cat# 229471) for enzymatic digestion using 0.5 mg/ml Collagenase IV (Gibco Cat# 17104-019), and 25 µg/ml DNase I (Thermo Scientific, Cat# J62229.MC) in AIM V media (5 ml per brain). Samples were incubated at 37°C while shaking (500 RPM) for 50 minutes. FBS was added to stop enzymatic activity, and cells were passed through a 100uM filter (Miltenyi, Cat# 130-098-463) and washed once with RPMI (Gibco, Cat# 21870-076 500ml) + 10% FBS (Omega Scientific, Cat# FB-12) and transferred to the new conical tube. Myelin was removed from the samples using a 37.5% Percoll gradient centrifugation as described previously (6, 11). The samples were resuspended in 1ml of 37.5% Percoll and then filled to the 7.5ml mark with more of the 37.5% Percoll. Tubes were slowly overlayed with 1X PBS up to the 10ml mark using a transfer pipette (Fisherbrand, Cat# 13-711-7M) before centrifuging for 25 minutes at room temperature at 2300 RPM with the brakes off. Widened 1000ul pipette tips (Biotix, Cat# 63305410) were used to remove the myelin layer and supernatant. The pellet was resuspended in 1ml of RPMI+ 10% FBS. and transferred to a new 15ml conical tube, filled up to 10ml with RPMI+ 10% FBS, and centrifuged for 5 minutes at 1800 RPM at 4°C. RBS lysis was performed using ACK lysis buffer 0.15M NH4CL (Sigma Aldrich, Cat# A-0171), 10MM KHCO3 (Fisher Scientific, Cat# P184-500), 0.1mM NaEDTA (Sigma Aldrich, Cat# E-1644). RBC lysis was stopped by restoring the osmolarity with PBS and 10% FBS. Cells were then stained for flow cytometry.

**Flow Cytometry**

Samples were stained at 4°C, protected from light, and washed with FACS buffer between steps. Cells were first incubated with Fixable Viability Dye eFluor™ 780 (ThermoFisher, Cat#65-0865-18) at 1:2000 dilution in PBS for 10 minutes. Fc receptors were blocked with TruStain FcX™ (anti-mouse CD16/32, Biolegend Cat# 101320) for 20 minutes then stained with following antibodies: VioletFluor™ 450 anti-mouse CD19 (Tonbo Biosciences, Cat# 75-0193-U100), BV480™ Rat anti-mouse I-A/I-E (MHCII) (BD Biosciences, Cat# 566086), BV 570™ anti-mouse Ly-6C (Biolegend, Cat# 128030), BV605™ anti-mouse/human CD11b (Biolegend, Cat# 101237), BV 650™ anti-mouse CD45.2(Biolegend, Cat# 109836), PE anti-mouse/human CD3e (Invitrogen, Cat# 12-0031-83), FITC Anti-Mouse CD4 (Tonbo Biosciences, Cat# 35-0041-U100), PerCP Anti-Mouse CD8a (Tonbo Biosciences, Cat# 67-0081-U100), PE/Fire™ 700 anti-mouse NK-1.1 (Biolegend, Cat# 108774), and Alexa Fluor® 700 anti-mouse/rat/human CD27 (Biolegend, Cat# 124240). UltraComp eBeads™ Compensation Beads (ThermoFisher Cat# 01-2222-42), ArC™ Amine Reactive Compensation Bead Kit (ThermoFisher, Cat# A10346) and cells from spleen served as controls for compensation and single stained samples for gating. Flow data was acquired on a Novocyte Quanteon flow cytometer, which was analyzed using Novocyte's NovoExpress software and FlowJo.

**Experimental autoimmune encephalomyelitis**

3 days after combined ICI treatment, mice were induced with active or adoptive transfer experimental autoimmune encephalomyelitis (EAE). Active EAE was induced via SQ injection containing 200 μg MOG_35–55_ peptide (MEVGWYRSPFSRVVHLYRNGK) emulsified in Complete Freund′s Adjuvant, supplemented with *Mycobacterium tuberculosis* H37Ra (Hooke Laboratories Cat #EK-0111) as previously reported (6). Briefly, SQ injection was given at a total dosage of 200 μl emulsion at two sites (100 μl per site) over the flank region. Additionally, on the day of EAE induction and 48 hours post-immunization, 200 ng pertussis toxin in PBS (List Biologic Laboratories, Cat. 181) was injected into each mouse (IP). To induce adoptive-transfer (AT) EAE, WT donor mice were first actively immunized with MOG_35-55_ peptide and a single dose of pertussis toxin as described previously (6, 11). 11 days later, cells were isolated from the inguinal draining lymph node (DLN) and spleen and were activated *in vitro* with 20 𝜇g/mL MOG_35-55_ peptide in the presence of 20 ng/ mL of rm IL-12 (p70, containing both p40 and p35 subunits) and 10 𝜇g/mL anti-IFNγ (Clone XMG1.2, BioLegend) for 72 hr to generate encephalitgogenic donor cells. ~20 x 10^6^ donor cells were injected (IP) into each WT recipient mice as described previously (6). Nutra-Gel wet food (Bio-Serve, Cat. S4798) was provided on the cage floor, along with long sipper bottles to facilitate feeding after the onset of EAE. Mice were carefully monitored daily to assess for any development of clinical signs using the following scoring system: 0 - no signs, 0.5 - partially limp tail, 1 - limp tail, 1.5 - limp tail and hind leg inhibition, 2 - hind limb paresis, 2.5 - one hind limb paralysis, 3 both hind limb paralysis, 3.5 - hind limb paralysis and weakness in forelimbs, 4 - tetraplegia, and 5 - moribund or euthanized due to severe paralysis (scored >3.5 for 2 consecutive days).

**Cytokine Quantification**

To assess cytokines, mice were sacrificed at 72 hours following the last combi-ICI/Vehicle injection. Blood (plasma) and brains (micro-dissected hippocampus) were collected for the cytokine ELISA. Brain tissue was homogenized in presence of protease inhibitors and centrifuged. Levels of cytokines in plasma and brain supernatants were analyzed using a magnetic bead-based multiplexed ELISA kit (Thermo Fisher Scientific).

**Statistical Analysis**

Statistical analyses were performed using one-way ANOVA to confirm overall significance (GraphPad Prism, v8.0, RRID:SCR_002798). All data are expressed as the mean ± SEM. For comparisons between the vehicle and ICI-treated groups for the cancer volume, non-parametric, two-tailed unpaired *t* tests were performed with Holm-Sidak’s correction. Two-way ANOVA with Tukey's multiple comparisons test was used to analyze ICI treatment in cancer and non-cancer groups. The Kruskal-Wallis test was used to analyze cytokine and IHC data. All analyses considered a value of *P* ≤ 0.05 to be statistically significant.

**RESOURCES**

| **REAGENTS or RESOURCES** | **SOURCE** | **IDENTIFIER** |
| --- | --- | --- |
| **Antibodies** | | |
| eBioscience™ Fixable Viability Dye eFluor™ 780 | ThermoFisher | Cat# 65-0865-18  RRID: N/A |
| Alexa Fluor® 700 anti-mouse/rat/human CD27 Antibody | Biolegend | Cat# 124240  RRID:AB_2810383 |
| BD Horizon™ BV480 Rat Anti-Mouse I-A/I-E | BD Biosciences | Cat# 566086, RRID:AB_2869739 |
| PE/Fire™ 700 anti-mouse NK-1.1 Antibody | Biolegend | Cat# 108774  RRID:AB_3083260 |
| PE anti-mouse/human CD3e Antibody | ThermoFisher | Cat# 12-0031-83  RRID:AB_465497 |
| PerCP Anti-Mouse CD8a (53-6.7) | Tonbo Biosciences | Cat# 67-0081, RRID:AB_2621882 |
| Brilliant Violet 421(TM) anti-mouse CD19 | Biolegend | Cat# 115549, RRID:AB_2563066 |
| Brilliant Violet 605(TM) anti-mouse/human CD11b | Biolegend | Cat# 101257, RRID:AB_2565431 |
| Brilliant Violet 650(TM) anti-mouse CD45.2 | Biolegend | Cat# 109835, RRID:AB_11203374 |
| FITC Anti-Mouse CD4 (GK1.5) | Tonbo Biosciences | Cat# 35-0041, RRID:AB_2621665 |
| Brilliant Violet 570™ anti-mouse Ly-6C Antibody | Biolegend | Cat# 128030, RRID:AB_2562617 |
| Rabbit anti IBA1 | FUJIFILM Wako | Cat# 019-19741; RRID: AB_839504 |
| Rat anti Mouse CD68 | Bio-Rad | Cat# MCA1957; RRID: AB_322219 |
| Mouse anti GFAP | FisherSci | Cat# MA5-12023, RRID:AB_10984338 |
| Rat anti BrdU | Abcam | Cat# ab6326, RRID:AB_305426 |
| Rabbit anti NeuN | Millipore | Cat# MABN140, RRID:AB_2571567 |
| Rabbit anti cFos | Abcam | Cat# ab190289, RRID:AB_2737414 |
| Mouse anti NeuN | Millipore | Cat# MAB377, RRID:AB_2298772 |
| Mouse anti Synaptophysin | Sigma | Cat# S5768; RRID: AB_477523 |
| Mouse anti PSD-95 | FisherSci | Cat# MA1-045; RRID: AB_325399 |
| Rat anti Myelin Basic Protein | Millipore | Cat# MAB386, RRID:AB_94975 |
| Donkey anti Rat AF 488 | FisherSci | Cat# A-21208, RRID:AB_2535794 |
| Goat anti Rabbit AF 488 | FisherSci | Cat# a11008; RRID: AB_143165 |
| Goat anti Mouse AF 568 | Abcam | Cat# ab175473, RRID:AB_2895153 |
| Goat anti Rat AF 568 | Abcam | Cat# ab175476, RRID:AB_2813739 |
| Goat anti Rat AF 647 | Abcam | Cat# ab150159; RRID: AB_2566823 |
| Goat anti Mouse AF 647 | Abcam | Cat# ab150115; RRID: AB_2687948 |
| Donkey anti Mouse AF 488 | FisherSci | Cat# A-21202, RRID:AB_141607 |
| Donkey anti Rabbit AF 568 | Abcam | Cat# ab175470, RRID:AB_2783823 |
| Anti-PD1 | BioXCells | Cat# BP0273 RRID: AB_2687796 |
| Anti-CTLA-4 | BioXCells | Cat# BP0032 RRID: AB_1107598 |
| Armenian Hamster IgG | BioXCells | Cat# BP0091, RRID: AB_1107773 |
| Rat IgG2a | BioXCells | Cat# BP0089, RRID: AB_1107769 |
| 10X DAPI | FisherSci | Cat# D1306, RRID:AB_2629482 |
| **Biological Samples** | | |
| D4M-UV2 Melanoma Cells | Dr. F. Maragonni | UC Irvine |
|  |  |  |
| **Chemical, peptides, and recombinant proteins** | | |
| 5-Bromo-2′deoxyuridine (BrdU) | Sigma | Cat# B5002 |
| Click-iT™ EdU | Invitrogen, | Cat# C10350 |
| Gibco™ DMEM (1X) + GlutaMAX™-I | FisherSci | Cat# 10-569-010 |
| Gibco^TM^ FBS | FisherSci | Cat# 10-082-147 |
| Gibco™ TrypLE™ Express Enzyme | FisherSci | Cat# 12-605-010 |
| Gibco™ Hibernate™-A Medium | FisherSci | Cat# A1247501 |
| Gibco™ DPBS (no magnesium, no calcium) | FisherSci | Cat# 14-190-144 |
| Gibco™ 10X DPBS (no magnesium, no calcium) | FisherSci | Cat# 14-200-075 |
| Nutra-Gel wet food | Bio-Serve | Cat# S4798 |
| MOG_35-55_/CFA Emulsion Immunization Kit | Hooke Laboratories | Cat# EK-0111 |
| Pertussis toxin | List Biologicals | Cat# 181 |
| Isospire™ (isoflurane) Inhalation Anesthetic | Dechra | N/A |
| Heparin sodium salt from porcine intestinal mucosa | Sigma | Cat# H3149 |
| Gibco™ AIM V™ Medium | FisherSci | Cat# 12055083 |
| Cytiva Percoll™ Centrifugation Media | FisherSci | Cat# 45001748 |
| Gibco^TM^ HBSS | FisherSci | Cat# 14-175-079 |
| Paraformaldehyde | Sigma | Cat# 158127 |
| Sodium Azide | Sigma | Cat# S2002 |
| Sucrose | Sigma | Cat# S7903 |
| O.C.T compounds | VWR | Cat# 25608903 |
| Normal Goat Serum (NGS) | Jackson ImmunoResearch Labs | Cat# 005-000-121, RRID:AB_2336990 |
| Normal Donkey Serum (NDS) | Jackson ImmunoResearch Labs | Cat# 017-000-121, RRID: AB_2337258 |
| VECTASHIELD® Antifade Mounting Medium | VectaShield | Cat# H-1000-10 |
| **Software and Algorithms** | | |
| Noldus EthoVisionXT 17 Tracking Software | Noldus | N/A |
| FreezeFrame | Coulbourn Instruments | N/A |
| FlowJo | Tree Star | N/A |
| Prism | GraphPad | N/A |
| Imaris 10.0 | Oxford Instruments | N/A |
| **Others** | | |
| 31 Gauge insulin syringe | BD Sciences | Cat# 328411-1 |
| Precision Glide Needle Only 18 Gauge, 1 1/2" | BD Sciences | Cat# 305195 |
| Electronic Digital Caliper | VWR | Cat# 62379-531 |
| 50 mL Falcon tubes | FisherSci | Cat# 14-432-22 |
| 15 mL Falcon tubes | FisherSci | Cat# 339650 |
| Whatman® Puradisc 25 syringe filters | Sigma | Cat# WHA67502502 |
| Noldus EthoVisionXT 17 Video Capture Hardware System | Noldus | N/A |
| LSRFortessa | BD Bioscience | N/A |
| Scientific Industries SI-1200 Enviro-Genie | Enviro-Genie Scientific | N/A |
| Nikon Eclipse Ti2 AX Laser scanning confocal microscope | Nikon | N/A |

**References for Supplemental Materials and Methods.**

1. Acharya MM, Baulch JE, Klein PM, Baddour AAD, Apodaca LA, Kramar EA, et al. New Concerns for Neurocognitive Function during Deep Space Exposures to Chronic, Low Dose-Rate, Neutron Radiation. eNeuro 2019;6
2. Barker GR, Bird F, Alexander V, Warburton EC. Recognition memory for objects, place, and temporal order: a disconnection analysis of the role of the medial prefrontal cortex and perirhinal cortex. J Neurosci 2007;27:2948-57
3. Barker GR, Warburton EC. When is the hippocampus involved in recognition memory? J Neurosci 2011;31:10721-31
4. Bourin M, Hascoët M (2003) The mouse light/dark box test. Eur J Pharmacol 463:55–65.
5. Milad MR, Quirk GJ. Fear extinction as a model for translational neuroscience: ten years of progress. Annual review of psychology 2012;63:129-51
6. Othy S, Jairaman A, Dynes JL, Dong TX, Tune C, Yeromin AV, Zavala A, Akunwafo C, Chen F, Parker I, Cahalan MD. Regulatory T cells suppress Th17 cell Ca2+ signaling in the spinal cord during murine autoimmune neuroinflammation. Proc Natl Acad Sci U S A. 2020 Aug 18;117(33):20088-20099.
7. Parihar VK, Allen BD, Tran KK, Chmielewski NN, Craver BM, Martirosian V, Morganti JM, Rosi S, Vlkolinsky R, Acharya MM, Nelson GA, Allen AR, Limoli CL (2015c) Targeted overexpression of mitochondrial catalase prevents radiation-induced cognitive dysfunction. Antioxid Redox Signal 22:78–91.
8. Parihar VK, Maroso M, Syage A, Allen BD, Angulo MC, Soltesz I, Limoli CL (2018) Persistent nature of alterations in cognition and neuronal circuit excitability after exposure to simulated cosmic radiation in mice. Exp Neurol 305:44–55.
9. Schubert I, Ahlbrand R, Winter A, Vollmer L, Lewkowich I, Sah R. Enhanced fear and altered neuronal activation in forebrain limbic regions of CX3CR1-deficient mice. Brain Behav Immun 2018;68:34-43.
10. Markarian M, Krattli RP, Jr., Baddour JD, Alikhani L, Giedzinski E, Usmani MT*, et al.* Glia-Selective Deletion of Complement C1q Prevents Radiation-Induced Cognitive Deficits and Neuroinflammation. Cancer Res **2021**;81:1732-44
11. Jairaman A, Othy S, Dynes JL, Yeromin AV, Zavala A, Greenberg ML*, et al.* Piezo1 channels restrain regulatory T cells but are dispensable for effector CD4(+) T cell responses. Sci Adv **2021**;7
